# Collembola cuticles and the three-phase line tension

**DOI:** 10.3762/bjnano.8.172

**Published:** 2017-08-18

**Authors:** Håkon Gundersen, Hans Petter Leinaas, Christian Thaulow

**Affiliations:** 1 Department of Mechanical and Industrial Engineering, Norwegian University of Science and Technology (NTNU), 7491 Trondheim, Norway; 2 Department of Bioscience, University of Oslo, Oslo, Norway

**Keywords:** springtails (Collembola), superhydrophobicity, three-phase line tension

## Abstract

The cuticles of most springtails (Collembola) are superhydrophobic, but the mechanism has not been described in detail. Previous studies have suggested that overhanging surface structures play an important role, but such structures are not a universal trait among springtails with superhydrophobic cuticles. A novel wetting experiment with a fluorescent dye revealed the extent of wetting on exposed surface structures. Using simple wetting models to describe the composite wetting of the cuticular surface structures results in underestimating the contact angles of water. Including the three-phase line tension allows for a prediction of contact angles in the observed range. The discrepancy between the contact angle predicted by simple models and those observed is especially large in the springtail *Cryptopygus clavatus* which changes, seasonally, from superhydrophobic to wetting without a large change in surface structure; *C. clavatus* does not have overhanging surface structures. This large change in observed contact angles can be explained with a modest change of the three-phase line tension.

## Introduction

Collembola, a group of small, terrestrial hexapods, have been known to possess remarkable water-repellent properties [1–7]. Robust water repellence has been the subject of extensive research, with naturally occurring surfaces providing the best known examples of this effect [8–9]. This effect has great potential for use in functional surfaces with effects like self-cleaning, drag reduction and air retention [10–12]. The field of superhydrophobic surfaces has made extensive use of biomimetic methods, where the imitation of natural surfaces provides the basis for artificial surfaces [9,13–14]. The exact nature of and the mechanism behind natural water-repellent surfaces is therefore of great interest beyond the field of biology. Many natural surfaces feature hierarchical structures, which are difficult to reproduce biomimetically. Collembola cuticles feature surface structuring on a single, sub-micrometer scale [5]; this makes Collembola cuticle structures easily reproducible, as well as more resilient against mechanical wear [7].

While the water repellency of Collembola has long been described in general, macroscopic terms, a specific mechanical explanation has been lacking. Cassie and Baxter described a composite wetting state, where water wets only the tops of surface features, without wetting the substrate in between [15]. The composite wetting state assumed by Cassie and Baxter is well known in a range of other natural superhydrophobic surfaces [9]. The stability of the composite wetting state on Collembola cuticles has been the subject of recent studies where surface features with overhanging geometries are presented as having a decisive role [4,7]. Such geometry occurs in several Collembola species [4] but is not a universal trait in these animals [5–6]. The presence or lack of overhanging surface features does not affect the apparent contact angles predicted by the Cassie–Baxter equation for a system in the composite wetting state, but does affect the stability of the composite wetting state. The apparent contact angle of a composite wetting state is predicted by the Cassie–Baxter equation, which underestimates the contact angle of Collembola cuticles [5] and sub-micrometer-sized surface structures in general [16]. The Cassie–Baxter equation also fails to predict changes in contact angle without an accompanying change in surface structure, such as the seasonal change in wetting characteristics for the Collembola species *Cryptopygus clavatus* [6].

The three-phase line tension (λ), or “line tension” for short, is an energy term associated with the line of contact between three phases (most commonly solid, water and air) in partially wetted systems [17]. In the case of a droplet of liquid resting on a solid surface, the three phase line is simply the contact line between the drop and the surface. In the case of a drop resting on the top of surface roughness features (i.e., a Cassie–Baxter model state), the three phase line is the sum of the contact lines of each wetted roughness top. By including a three-phase contact line term in the equation for the apparent contact angle, Zheng et al. [16] were able to predict the size-scale dependency of the apparent contact angle for sub-micrometer surface structures. The effect of the three-phase line tension on the apparent contact angle is significant for systems with a large three-phase line length relative to the wetted surface area. An example of such a system is the wetting of the micrometer- and sub-micrometer-sized cuticular granules of Collembola. We propose that by using the equation of Zheng et al. [16] the high contact angles observed in Collembola can be predicted in general, and also a possible mechanism for the seasonal change of *Cryptopygus clavatus* in specific can be provided.

**Figure 1:**
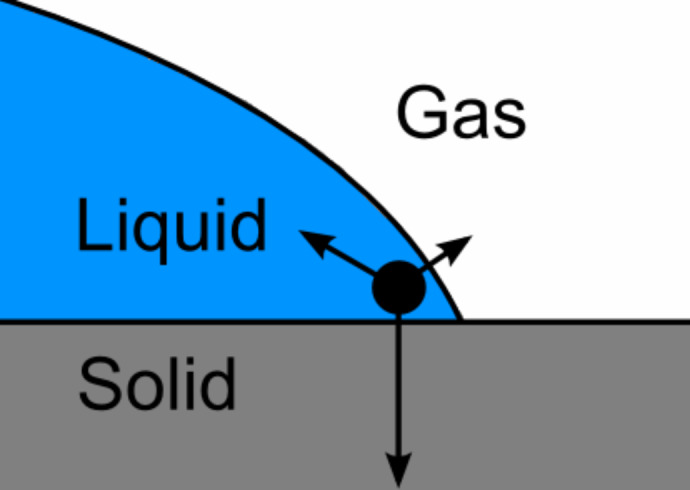
Imbalance of intermolecular forces for molecules near the three-phase contact line. The length of the arrows indicate the relative strength of the intermolecular interactions with the different phases. Adapted from Amirfazli and Neumann [17].

The molecules near three-phase contact lines are subjected to different intermolecular forces, compared to molecules in bulk phases, which results in a line tension. This is analogous to how the balance of intermolecular forces acting on a molecule near a two-phase interface result in an interfacial tension, see Figure 1. The value of the line tension remains debatable with reported experimental magnitudes ranging from λ = 10^−5^ N to λ = 10^−11^ N [17–18]. Theoretical models predict lower magnitudes for the line tension, Marmur predicted an upper limit of λ *<* 5 × 10^−9^ N [19], Bormashenko considered a values in the range λ = 10^−9^ N to λ = 10^−12^ N to be realistic [18], and de Gennes reported λ = 10^−11^ N [20]. A majority of experimental studies on solid–liquid–vapor systems fall in the higher end of the range, the differences between the experimental results and the theoretical predictions can likely be explained by contamination of the solid surfaces or experimental error [17–18,20]. Pompe et al. accounted for substrate inhomogeneities with local high-resolution imaging at the contact line and reported values in the range of λ = 10^−10^ N [21]. There is also contention with regards to the sign of the line tension [17–18], several theoretical studies predict both negative and positive signs for the line tension [19], while a majority of experimental studies of solid–liquid–vapor systems report a positive sign [17]. Despite the debate with regards to the exact value of the line tension it remains an important concept for surfaces with sub-micrometer roughness features because such small size scales result in very long total three-phase line lengths and correspondingly large total line energies.

Partial wetting is a state where a liquid rests on top of surface roughness features such that the roughness tops are wetted, while the substrate between tops is not wetted. Partial wetting is often referred to as a composite wetting state or a Cassie state after the early proposed equation of Cassie and Baxter (Equation 1) based on a surface-energy argument for the case of a droplet resting on a composite surface [15],

[1]
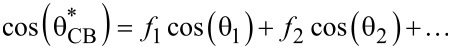


where θ^*^ is the apparent contact angle, *f_i_* is the fraction of the surface *i* in contact with the water drop and θ*_i_* is the inherent contact angle of a smooth surface *i*. For the special case of a water drop on the roughness tops of a chemically homogeneous rough surface (*i* = 1) in air (*i* = 2), Equation 1 is reduced to Equation 2 through the assumptions that *f*_1_ + *f*_2_ = 1 and cos(θ_2_) = −1,

[2]
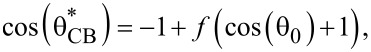


where *f* is the solid area fraction, θ_0_ is the inherent contact angle of the smooth solid.

Zheng et al. included a the three-phase line tension in the Cassie–Baxter equation, see Equation 3 [16],

[3]
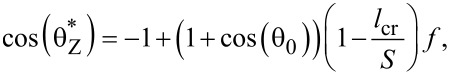


where *S* is the “roughness factor” *S* = *A*_s_/*L*, the ratio between the cross-sectional area (*A*_s_) and perimeter (*L*) of a surface roughness top. *l*_cr_ is an “intrinsical chemical length” given by Equation 4,

[4]
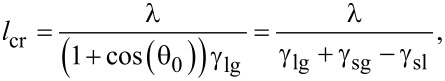


where λ is the three-phase line tension and γ_lg_, γ_sg_ and γ_sl_ are the interfacial energies of the liquid–gas, solid–gas and solid–liquid interfaces, respectively.

Equation 3 can be written in an equivalent form without the novel parameters (*S* and *l*_cr_), see Equation 5.

[5]
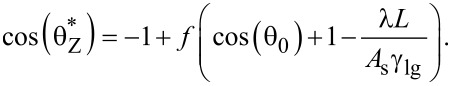


This form of the equation is very similar to the equation proposed by Wong and Ho [22]. The Zheng equation includes the dependency of the total three-phase line length on the cross-sectional shape of the surface roughness features, for the case of circular cross sections the two are identical.

Bormashenko presented a general equation for the wetting of rough, chemically homogeneous surfaces

[6]
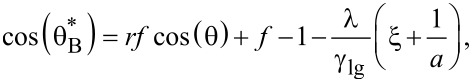


where *r* is the roughness ratio of the wetted area, ξ is the perimeter of the triple line per unit area of the substrate under the droplet, and *a* is the radius of the droplet [23]. This equation comprises both the Cassie–Baxter equation (Equation 2), when *r* = 1 and the effect of the line tension is negligible, and the Zheng equation (Equation 5) as well as the equation presented by Wong and Ho [22], when *r* = 1 and the effect of the internal three-phase contact line greatly exceeds that of the external contact line (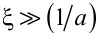).

Zheng et al. [16] developed Equation 3 to describe the case of droplets resting on top of small surface roughness features, treating the three-phase line tension as a parameter to describe different contact angles observed for surfaces with feature tops with different size scales but identical solid area fraction. Zheng et al. reported that a line-tension magnitude of 1.57 × 10^−8^ N was found to provide a good agreement between results and theory for roughness scales down to *S* ≈ 0.3 μm, below which the equation predicts contact angles of 180°.

**Figure 2:**
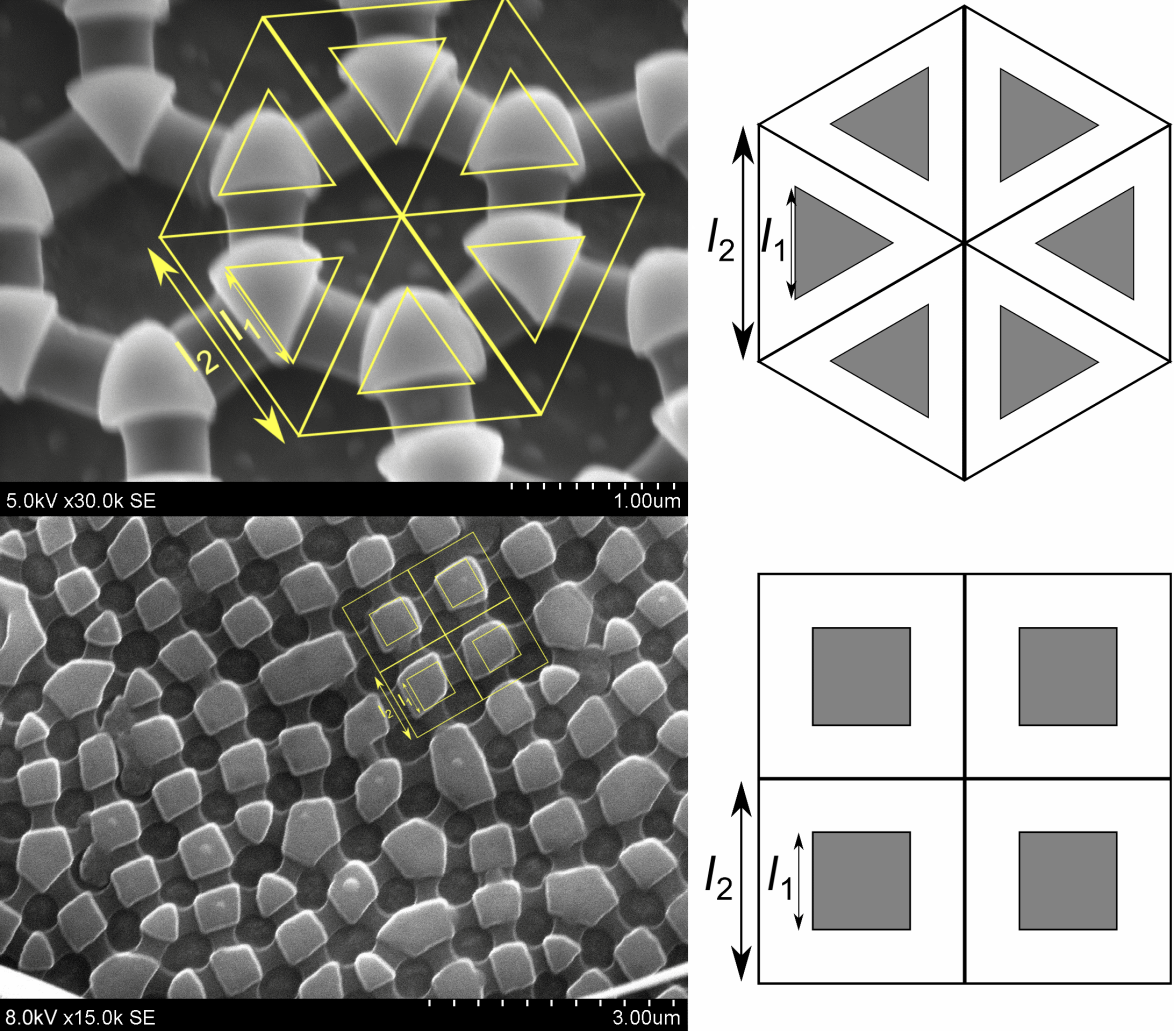
A simple tessellating pattern that can approximate Collembola cuticles for the partial wetting state where only granule tops are wetted. The two characteristic lengths (*l*_1_ and *l*_2_) can be used to determine the relevant parameters 
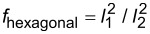
, 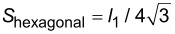, 
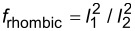
 and 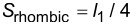. The geometric patterns are overlaid in SEM images of *Xenylla maritima* (hexagonal) and *Cryptopygus clavatus* (rhombic) for comparison.

Certain assumptions or simplifications must be used in order to apply these equations to Collembola cuticles. Thicker and thinner parts of the Collembola cuticles form recognizable patterns. The thicker parts are referred to as granules, and are connected by sections of intermediate thickness referred to as ridges. These granules are of sub-micrometer size, usually in the range of a few hundred nanometers, and typically form a hexagonal pattern of triangular granules connected by straight ridges [24]. A rhombic pattern of rhombic granules is also common [5,24–25], this is the type of pattern on our focal species *Cryptopygus clavatus* [6]. The partial wetting state where only granule tops are wetted can be approximated by simple tessellating patterns. The repeating unit is a three-sided prism, surrounded by a triangular open space, for approximately hexagonal cuticle patterns. For approximately rhombic cuticle patterns, the repeating unit is a four-sided prism, surrounded by a square open space. See Figure 2 for a comparison of the hexagonal and rhombic approximations. The two characteristic lengths (*l*_1_ and *l*_2_) can be used to determine the relevant parameters 
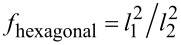
, 
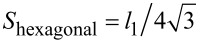
, 
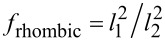
 and 
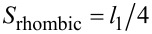
.

Both the Cassie–Baxter model and Zheng’s models of wetting, include the inherent contact angle of the substrate (θ_0_) as a parameter. Substrates with θ_0_ *<* 90° are not expected to form stable composite wetting states, which is a prerequisite of both models. Surface structures with reentrant geometry (overhang), present in some Collembolan species [4], can support composite wetting states with any value of θ_0_ [26], but such structures are not a universal trait in these animals [5–6]. The upper limit of θ_0_ is about 120° for real surfaces, observed on perfluorinated polymers, or 156 ° for a theoretical surface with no surface tension [27]. Insect waxes fall in the range of 90–110 °, typically around 105° [28–29]. The range of reasonable values for θ_0_ is therefore limited to 90–120°, where the lower bound is a prerequisite of the composite wetting state and the upper bound is the highest known value for real surfaces.

The model for predicting the apparent contact angles becomes dependent on the size scale when the area-to-perimeter ratio (*S*) of surface features is included. The magnitude of the size-scale dependency is determined by the three-phase line tension (λ). The exact magnitude of λ is not known for the Collembola cuticle, water, air three-phase system. Zheng’s model can either be used with measured contact angles to estimate λ or with estimated values of λ to predict the apparent contact angle θ^*^ of systems with known geometry. For low values of λ the contact angles predicted by Equation 5 approach that of the Cassie–Baxter model (Equation 2), which means that *f* is the dominant factor, high values of λ give 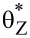 = 180°. Exactly what constitutes “low” and “high” values of λ is determined by Zheng’s “roughness factor” *S*, as an example *f* = 0.25 and *S* = 0.1 μm predicts a contact angle within 0.5° of that of the Cassie–Baxter model for λ *<* 10^−10^ N, while 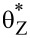 = 180° is predicted for λ *>* 10^−8.27^ N.

**Figure 3:**
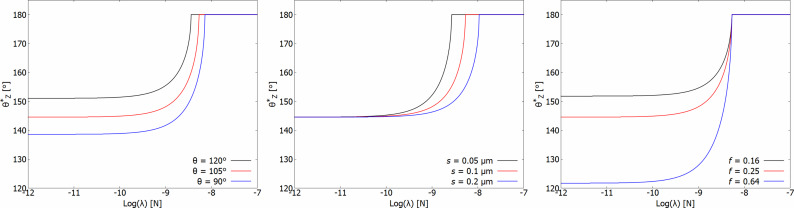
The effect of θ_0_, *S* and *f* on the model of Zheng et al. [16] for 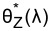. A system with θ_0_ = 105°, *S* = 0.1 μm and *f* = 0.25 is shown as a red line in each graph.

Figure 3 shows the Zheng model 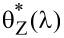 for a system with θ_0_ = 105°, *S* = 0.1 μm and *f* = 0.25 as the red line in each of three graphs where the effect of varying the inherent contact angle (θ_0_), the roughness factor (*S*) and the solid area fraction (*f*) are demonstrated. Small values of λ yield a result that approaches that of the regular Cassie–Baxter model (a horizontal line), while high values of λ result in a prediction of perfect non-wetting (shown as 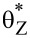 = 180°). Between the extremes of pure Cassie–Baxter behavior and pure non-wetting a critical range of λ is found, where the exact magnitude of λ determines the contact angle. Changing the roughness factor shifts this critical range of λ, but does not qualitatively change the behavior. Changing the solid area fraction changes the value predicted by the Cassie–Baxter model, and thus shifts the minimum value of 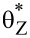 for low values of λ. Changing the inherent contact angle shifts both the minimum value of 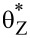 and the critical range of λ.

We propose that the composite wetting state assumed by the Cassie–Baxter model, as well as the derivative Zheng model, can be demonstrated by a novel wetting experiment with a dye. Nickerl et al demonstrated a lipid layer (epicuticular wax) covering all parts of the Collembola cuticle, using time-of-flight secondary ion mass spectrometry [30]. A lipophilic dye, such as Nile Red, will bind to any part of such a layer it came into contact with, thus staining the part of a surface wetted by the dye. The parts of the cuticle that were wetted by the dye can then be visualized with fluorescence microscopy.

## Results and Discussion

**Figure 4:**
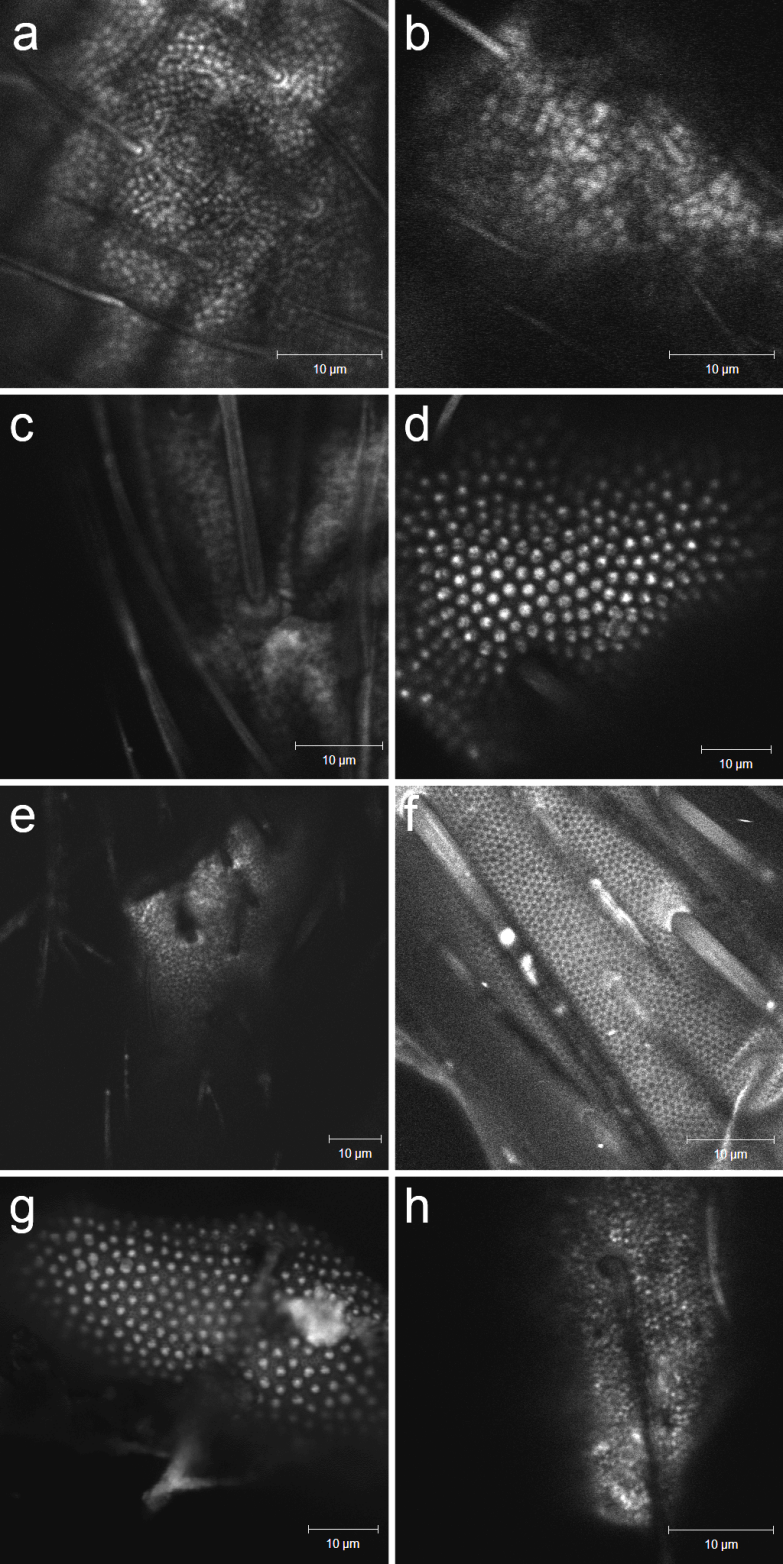
Stained samples imaged with fluorescence microscopy, showing the tops of primary and (present in d and g) secondary granules. The light areas are those where the lipophilic dye has bonded with the surface, indicating wetting contact between the dye solution and a lipid layer. The images were obtained with confocal fluorescence microscopy using incidental light of 488 nm wavelength and a bandpass filter (565–615 nm). a) *C. clavatus* (winter-acclimated), b) *C. clavatus* (summer-acclimated), c) *F. quadrioculata*, d) *H. viatica*, e) *I. prasis*, f) *O. flavescens*, g) *Onychiurus sp.*, h) *P. flavescens*.

Collembola cuticles were dyed with a water–acetone solution of Nile Red and imaged with fluorescence microscopy, a selection of cuticles are shown in Figure 4. The tops of primary and secondary granules are clearly visualized, the base between granules was not visualized on any samples. This indicates that the tops of the granules were wetted by the dye solution, while the base cuticle was not wetted. This is in accordance with the assumption of a composite wetting state, with a wetted area fraction (*f*) corresponding to the area fraction of cuticular granules.

The area fraction covered by granules (*f*) is the main parameter used to estimate apparent contact angles by the Cassie–Baxter equation (Equation 2). Nickerl et al. [25] studied the cuticle structure of a larger selection of Collembola. The geometric measurements (granule size and distance) can be used to estimate the area fraction covered by granules (*f*) for species with regular granule patterns, figure Figure 2 shows such an approximation for rhombic and hexagonal granule patterns. We applied this estimation method to the measurements of Nickerl et al., which yielded a range of granule area fractions from 0.111 to 0.709, compared to a range of 0.137 to 0.697 from a reassessment of our measured values published in [5] by the same method of estimation. The selection of species by Nickerl et al. covered all orders of Collembola (Entomobryomorpha, Poduromorpha, Symphypleona, Neelipleona). In comparison, in our previous study [5], we selected species from habitats ranging from extremely dry to very wet in order to obtain a wide diversity of Collembola surface structures [31–32]. Since all orders of Collembola and a wide range of surface structures and habitat types are considered in these two studies, it seems likely that the granule area fraction of most Collembola will fall within the two extremes of 0.111 to 0.709. If Equation 2 is used to estimate the contact angle of this range of values of *f*, the resulting range is 118–157°.

Direct measurement of the contact angles of Collembola cuticles are scarce, but their wetting behavior is variously described as “non-wetting” [30,33] “anti-wetting” [2,33] and “unwettable” [1]. The common classification of “superhydrophobic” surfaces requires an apparent contact angle exceeding 150°, and a contact angle hysteresis no larger than 10°. The predicted contact angles for all but the lowest values of *f* (and consequently highest values of 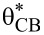) yielded by the Cassie–Baxter equation do not reflect the observed apparent contact angles of most Collembola cuticles. The authors [5] previously found that the Cassie–Baxter equation systematically underestimated the contact angle, compared to measured values.

**Figure 5:**
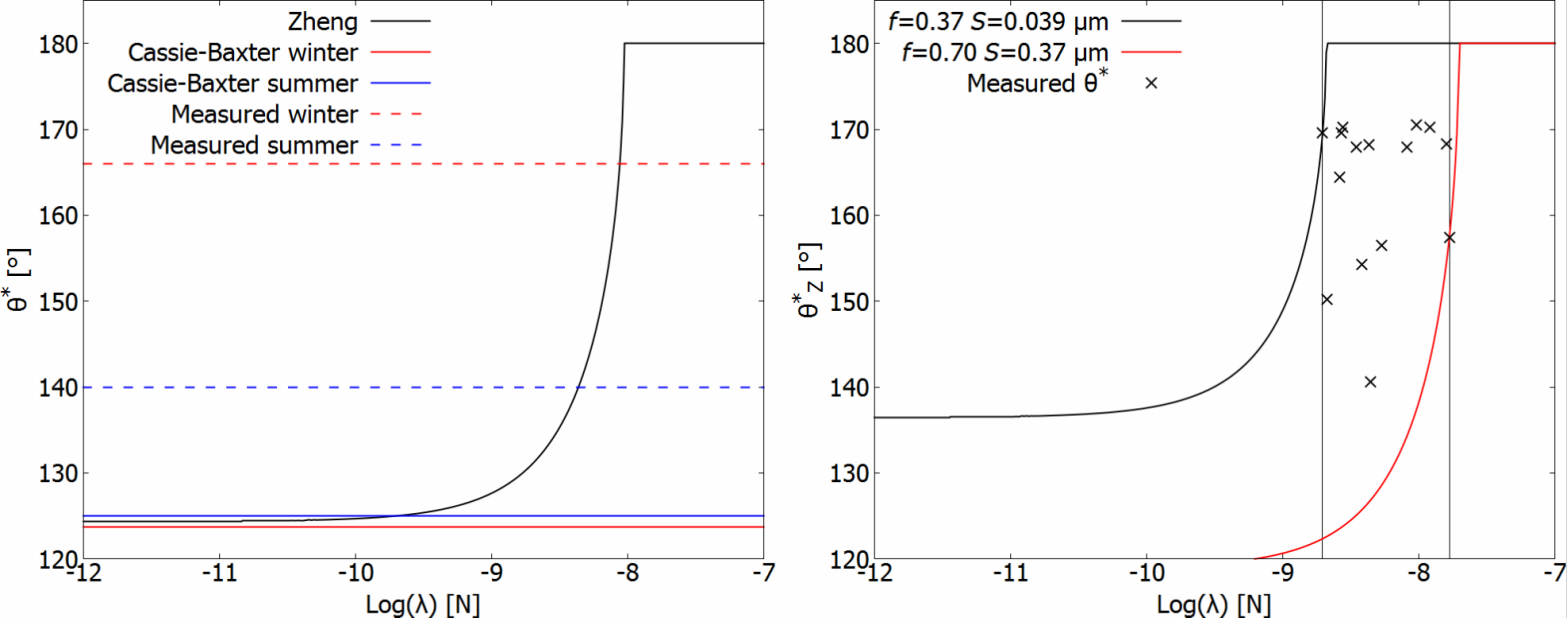
Left: Measured and predicted apparent contact angles (θ^*^) as a function of log(λ) for *C. clavatus* acclimated in captivity to winter- and summer-like conditions. Solid lines in red and blue show the predictions of the Cassie–Baxter model, dashed lines in red and blue the measured values, and the solid black line shows the prediction of the Zheng model. Assumptions: θ_0_ = 105°, *f* = 0.588, *S* = 0.176 μm, based on results described in [6]. Right: 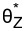 as a function of log(λ) for the two combinations of *f* and *S* that yield the highest and lowest estimated values of λ found among species in Gundersen et al. [5] and with estimated values of λ noted for each species.

The Zheng model (Equation 3) includes the roughness parameter *S* and the three-phase line tension λ. *S*, the ratio of three-phase contact line length and wetted surface area, can be calculated from surface structure data. A reassessment of measured values published in [5] yielded a range of *S* from 0.039 μm to 0.37 μm in the Collembola species studied. λ, the three-phase line tension, of water on Collembola cuticles, or similar systems, is not known. There are two possible approaches, assume a single value of λ for all Collembola species studied, and use it to predict apparent contact angles. Alternatively, assume that λ can vary from one species to another and use measured values of the contact angle to estimate reasonable values of λ. Estimated values of λ for each species are shown in Figure 5, right panel, where *f* and *S* are based on the reassessment of data from [5] and θ_0_ = 105° was assumed. All intersections between the observed contact angle θ^*^ and the apparent contact angles predicted by the Zheng model 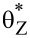 are marked, while the sets of *f* and *S* that mark the upper and lower bounds for 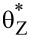 for the studied species are shown as solid lines. All estimates of λ where found in the range from λ = 2 × 10^−9^ N to λ = 2 × 10^−8^ N; the values are summarized in Table 1. This is within the range of published values for three-phase line tension in vapor–liquid–solid systems, but exceeds the values predicted by theoretical studies [17–18].

**Table 1:** Estimated values of the three-phase line tension (λ). Estimates are based on primary granules, unless otherwise noted.

species	*S* [μm]	*f*	λ [N]	notes	ref.

*Anurophorus laricis*	0.37	0.70	1.68 × 10^−8^		[5]
*Anurophorus septentrionalis*	0.32	0.60	1.60 × 10^−8^		[5]
*Archisotoma besselsi*	0.039	0.37	1.97 × 10^−9^		[5]
*Archisotoma besselsi*	0.061	0.14	2.71 × 10^−9^	based on secondary granules	[5]
*Cryptopygus clavatus*	0.17	0.59	4.40 × 10^−9^	summer-acclimated	[5]
*Cryptopygus clavatus*	0.18	0.58	4.21 × 10^−9^	summer-acclimated	[6]
*Cryptopygus clavatus*	0.18	0.60	8.82 × 10^−9^	winter-acclimated	[6]
*Desoria oliviaca*	0.059	0.30	2.62 × 10^−9^		[5]
*Folsomia quadrioculata*	0.19	0.37	9.67 × 10^−9^		[5]
*Hypogastura viatica*	0.070	0.50	3.50 × 10^−9^		[5]
*Hypogastura viatica*	0.19	0.15	8.16 × 10^−9^	based on secondary granules	[5]
*Isotoma anglicana*	0.12	0.33	3.82 × 10^−9^		[5]
*Isotomurus prasis*	0.095	0.19	4.33 × 10^−9^		[5]
*Onychiurus sp.*	0.054	0.38	2.78 × 10^−9^		[5]
*Onychiurus sp.*	0.24	0.30	1.21 × 10^−8^	based on secondary granules	[5]
*Orchesella flavescens*	0.080	0.35	2.11 × 10^−9^		[5]
*Xenylla maritima*	0.15	0.33	5.32 × 10^−9^		[5]

The Collembola *Cryptopygus clavatus* changes between superhydrophic water repellance with plastron formation upon submersion under winter conditions and active grazing underwater with no visible plastron under summer conditions [6]. This change in wetting behavior is not accompanied by considerable structural changes in the cuticle. Gundersen et al. concluded that changes in the epicuticular wax layer was a possible explanation. Assuming θ_0_ = 120° in Equation 2 yields a predicted contact angle of 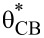 ≈ 135°, below the contact angle observed in both summer- and winter-acclimated animals (Figure 5). The coverage of epicuticular wax was previously assumed to be either the top of the cuticular granules, leaving the areas between the granules exposed, or the entirety of the cuticle, recent studies conclude that the entire cuticle is covered [1–2,30]. In a wetting model that assumes contact only at the top of the cuticular granules these two extents of coverage give the same result. A study with a lipophilic dye is not suited to differentiate between the two, but would have revealed any loss of wax coverage on the top of granules upon summer acclimation. Figure 4 shows stained samples of winter- and summer-acclimated *C. clavatus* under fluorescing conditions. The immediate conclusion is that the top of the granules are covered in epicuticular wax in both the winter- and summer-acclimated state, and that changes in the extent of the wax layer can not explain the seasonal change in wetting behavior.

Figure 5, left panel, shows the discrepancy between measured values of the apparent contact angle (dashed red and blue lines) of *C. clavatus* and the predictions of the Cassie–Baxter model (solid, red and blue lines) along with the Zheng model (solid black line). Values for *f* and *S* were based on assessment of SEM images from Gundersen et al. [6], where a small seasonal change in the physical structure was observed. This change in surface structure results in a change in the predicted values of the Cassie–Baxter model (solid, red and blue lines) that is much smaller than the change in observed values (dashed, red and blue lines). The intersect between the Zheng model (described by Equation 3) and the measured values give estimates for the value of λ on *C. clavatus*, λ = 8.82 × 10^−9^ N for winter-acclimated animals and λ = 4.21 × 10^−9^ N for summer-acclimated animals. This modest change in the magnitude of the three-phase line tension can explain the seasonal change in wetting characteristics found in *C. clavatus* from summer to winter adaption, without large structural changes in the cuticle. Collembola are covered in an epicuticular wax layer, which is supplied through pores in the cuticle. Collembola also molt, which changes the entire epicuticle. It is possible for Collembola to change the chemical composition of the wax layer, either gradually through the cuticular pores, or upon molting, when the entire layer is replaced.

## Conclusion

The very large apparent contact angle of water on Collembola cuticles can not be predicted by the conventional wetting models [5]. The parameters in these models, wetted area fraction (*f*) and inherent contact angle (θ_0_), can vary within a certain range, but not enough to explain the observed contact angles. The wetted area fraction can be demonstrated by experimental methods, as shown here, as well as through mathematical modeling of the energy needed to transition from composite wetting to non-composite wetting [4]. This yields a wetted area that is determined by the area covered by cuticular granules, which for Collembola constitutes a range from 0.111 to 0.709 in the work of Nickerl et al. [25], and a range of 0.137 to 0.697 in a reassessment of data from our previous work [5]. The Cassie–Baxter model [15] greatly underestimates the apparent contact angles of these cuticles for most of this range of *f* compared with the measured values of water on Collembola cuticles. The inherent contact angle, while theoretically ranging from 0 to 156° [27], is limited to a reasonable range of 90° (the minimum for a stable composite wetting state) to 120° (the highest known for a smooth solid). This range of inherent contact angles is not sufficient to explain the range of observed apparent contact angles. The assumption for Collembola cuticles in this work was θ_0_ = 105°, which corresponds to that of many insect waxes [28–29]. The model for predicting apparent contact angles becomes scale-dependent when the three-phase line tension (λ) is considered [16]. In the specific case of Collembola, the size scale is of a magnitude where the three-phase line tension can explain the discrepancy between the observed apparent contact angles and those predicted by classical models. A three-phase line tension in the range from 2 × 10^−9^ N to 2 × 10^−8^ N can account for this difference, this is within the range of previously reported experimental values of the three-phase line tension, but exceeds the values predicted by theoretical studies [17–18].

## Experimental

Springtails are non-regulated invertebrates and not subject to animal experiment laws in Norway. The species studied are not endangered or protected. The animals were collected in the wild in Norway in public areas with no restrictions on the gathering of invertebrates. The animals were killed with chloroform vapor immediately before experiments.

Nile Red dye was dissolved in acetone to form a stock solution at 1 mg/mL. This was further diluted 1:100 with 10 vol % acetone (aq) to form an aqueous acetone dye. Samples were soaked in the dye solution for 5 min and subsequently rinsed with acetone and air-dried.

Samples were studied with a Zeiss 510 confocal laser scanning microscope. Fluorescense microscopy was performed with incidental light of 488 nm wavelength and a bandpass filter (565–615 nm). Reflected-light microscopy used a bandpass filter (480–520 nm). All imaging was done with a water-immersion objective, with the samples immersed in purified water. The cuticle (including granules and ridges) of the dorsal metasoma was studied.

Some unstained arthropod cuticles will autofluoresce, this was observed for several of the studied species: *C. clavatus*, *F. quadrioculata*, *H. viatica*, *I. prasis* and *Onychiurus sp.* Setae, rings in the cuticle around the base of setae, primary and secondary granules were imaged in fluorescent lighting on unstained samples (not all features were equally autofluorescent on all studied species). This autofluorescence effect can easily be distinguished by its weaker luminescence. On average, a light intensity one order of magnitude higher was required to visualize features based on autofluorescence alone, as compared to stained samples.

Samples were mounted on SEM stubs with silver glue and imaged with no applied conductive layer (i.e., no metalization or carbon coating). An FEI Quanta FEG 450 ESEM was used, utilizing the large field-of-view detector, which detects a combination of secondary and back-scattered electrons, in the low-vacuum mode. Typical imaging settings were 0.50 mbar (water vapor) chamber pressure, *E*_acc_ = 10 kV.
